# “My partner was not fond of using condoms and I was not on contraception”: understanding adolescent mothers’ perspectives of sexual risk behaviour in KwaZulu-Natal, South Africa

**DOI:** 10.1186/s12889-020-08474-2

**Published:** 2020-03-18

**Authors:** Desiree Govender, Saloshni Naidoo, Myra Taylor

**Affiliations:** 1 KwaZulu-Natal Department of Health, Durban, South Africa; 2 School of Nursing and Public Health, Discipline of Public Health Medicine, University of KwaZulu-Natal, Durban, South Africa; 3 Developing Research Innovation Localisation and Leadership (DRILL), Durban, South Africa

**Keywords:** Adolescent pregnancy, Sexual risk behaviour, Transactional sex, HIV, Sexual and reproductive health

## Abstract

**Background:**

Adolescent pregnancy has been a persistent area of interest and concern in the field of public health. The debate about adolescents’ sexual risk behaviour has also gained prominence due to findings that have demonstrated that adolescent girls between 15 and 19 years of age give birth to 16 million infants and account for 62% of new HIV infections in the Caribbean and African regions. Health compromising behaviours often develop in adolescence, yet the sexual and reproductive health of adolescent mothers is often marginalised in the healthcare field. The aim of this study was to explore adolescent mothers’ understanding of sexual risk behaviour.

**Methods:**

The study employed a descriptive qualitative design. To collect the data, four focus group discussions were conducted with adolescent mothers aged 16–19 years. The eighteen adolescent mothers were recruited using purposive sampling technique from a hospital in the Ugu district in KwaZulu-Natal, South Africa. Data were analysed using thematic analysis.

**Results:**

The study revealed that decisions to engage in risky sexual behaviour is influenced by peer pressure, drugs and alcohol, sexual experimentation, myths about contraception, the media, poor parental supervision and power gender dynamics, poverty leading to transactional sex, the vulnerability of young girls, and the fear of partner rejection.

**Conclusion:**

The findings of this study will contribute to a better understanding of adolescent mothers’ perspectives of sexual risk behaviour. In the subject matter of sexual and reproductive health, adolescents’ autonomy with respect to cultural and social recommendations should not be sidelined. Due to their vulnerability, adolescent women are exposed to transactional sex, and it is particularly due to poverty that adolescent women are driven into sexual relations with older men as a means of survival. Moreover, interventions to curb postpartum sexual risk behaviour are important to protect adolescent women and mothers against HIV/AIDS. The sexual and reproductive education of adolescent women should focus on resilience, negotiating skills, and protective decision making. Collaborative efforts to curb sexual risk taking by young women should be encouraged and should involve relevant agents from the educational, social and clinical fields.

## Background

Adolescent pregnancy has been a major area of interest in the field of public health [1] and the issue of adolescent childbearing has received critical attention due to the increased risk of adverse maternal and neonatal outcomes [1]. Furthermore, the debate about adolescents’ sexual risk behaviour has gained prominence as findings have demonstrated that adolescent girls between 15 and 19 years of age account for 62% of new HIV infections and this group gives birth to 16 million infants in the Caribbean and African regions [2]. Of the two million adolescents living with HIV, 82% live in sub-Saharan Africa. On a daily basis, 460 adolescent girls become infected with HIV in eastern and southern Africa [3] South Africa has the largest prevalence of HIV (13.5%, 7.97 million people living with HIV) in the world, and early adolescent pregnancies in South Africa are also associated with a high risk of acquiring HIV [4, 5]. As the HIV epidemic escalates in South Africa [4], adolescent pregnancy is a critical issue as it demonstrates the ineffectiveness of public health efforts to promote safe sexual behaviour. Other multifactorial factors that contributes to adolescent pregnancy also includes under resourced healthcare systems, stock out of contraceptives and staff burnout.

Health compromising behaviours often develop in adolescence, yet the sexual health of adolescent mothers is often marginalised [6]. The consideration of the natural stages of adolescence which fosters risk taking behaviours is important. Risk taking behaviours reflects the developmental changes in the brain [7]. As children progress to the stages of adolescence, intense brain changes occur which affects thinking and behaviour. Two primary brain functions (social-emotional system and the cognitive control system) matures at different rates [7, 8]. The social-emotional system, involving the amygdala matures more rapidly than the prefrontal cortex (cognitive control system [7, 8]. As the prefrontal cortex is still developing, adolescents may make decisions and solve problems guided by the amygdala, a portion of the brain responsible for emotions and emotional behaviours, leading to increased reward seeking [8]. Therefore, risk-taking behaviour is driven by the social-emotional processes. Increased reward –seeking behaviour and poor impulse control, at this stage makes adolescents vulnerable to problems related to sexual behaviours [7]. Health behaviours that develop during adolescence may shape adulthood but this period of adolescence is disregarded by researchers and policy makers as an important stage of human development [9].

Traditional approaches to adolescent sexual and reproductive health education place a high premium on the individual, whereas each individual is part of an ecosystem where biological changes, the influence of family and friends, community practices and behaviours, and access (or lack of access) to economic and academic opportunities play an important role in their lives [10]. Adolescents depend on their families, friends, peers, communities, schools, and health service personnel to acquire skills that can assist them in their transition from childhood to adulthood [11]. The social determinants of sexual and reproductive health can therefore only be transmitted through strong partnerships and collaboration among the education, social and health sectors [11].

The negative consequences and social stigma associated with adolescent childbearing may result in depression and low self-esteem [12, 13]. It is often against this backdrop that adolescent mothers engage in risky sexual behaviours, and this creates increased possibilities for repeat pregnancies regardless of the challenges that may have been experienced during the first pregnancy [14–18]. Moreover, pregnant and parenting adolescents are vulnerable to HIV infection due to a lack of resources, social status, and power [5, 15, 16]. Much of the literature on the sexual risk behaviour of pregnant adolescents and adolescent mothers suggests that these young women are negatively inclined towards contraception and condoms [15, 17, 18]. Factors that exacerbate high risk sexual behaviour include dysfunctional family structures, poor parental supervision, alcohol and drug use/abuse, peer pressure, a history of child abuse, psychological distress, intimate partner violence, poverty, gender power relationships, and poor contraceptive practices [1, 15, 19–24].

Recent statistics in South Africa have revealed that the birth rate among adolescents is 71 births per 1000 girls in the age category 15–19 years [25]. In an investigation into the phenomenon of adolescent repeat pregnancy, Govender et al. [18] found that the prevalence was 19.9% in KwaZulu- Natal, South Africa. Disturbingly, adolescent mothers who experienced a repeat pregnancy had a high rate of HIV infection and many were involved with partners that were 5 years or older than they were [18]. Adolescent repeat pregnancy is undeniably a symptom of the growing problem of high risk sexual behaviour among previously pregnant adolescent girls and mothers [18]. Patriarchy and gender power imbalances in South Africa have been recorded as influential in the sexual behaviour of and the relationships that young women forge [26–29]. It is therefore a concern that the most significant public health threat to curbing HIV infection in South Arica seems to be the romantic relationships and transactional sex that are prevalent between older men and younger women [30, 31].

According to UNAIDS [3], seven in 10 adolescent women have limited knowledge about HIV in sub-Saharan Africa. Sexual education programmes are sidelined and restricted [3]. Adolescents also have limited knowledge on preventing a pregnancy and there is also the reluctance of nurses and educators to provide SRH services and sexuality education. Furthermore, the gender norms and culture is also associated with taboos about sexual and reproductive health (SRH), seeking SRH services and making informed decisions about SRH [3]. Many adolescent girls in sub-Saharan Africa do not have access to modern methods of contraception. Furthermore, in the literature, a South African study by Singh and Naicker on sexual risk amongst young mothers aged 16 to 19 years, revealed that young mothers acknowledged that they were advised by their mothers on “how to not become pregnant” (p.167) but this advice did not include contraception use [32].

Many studies on adolescent pregnancy and parenting have been written by South African experts in the education, social and anthropology spheres drawing on individuals’ experiences of this phenomenon [32–42]. There has also been a proliferation of studies regarding the gender, sexuality and social aspects of adolescent pregnancy and parenting [33–42]. The educational and social effects of early motherhood is important. However, adolescent pregnancy and parenting is not confined to the education sector as public health professionals witness the complexities associated with this phenomenon.

The public health approach has evolved with time shifting its focus from disease to lifestyles and behaviours [43]. Sexual and reproductive health behaviour is an important aspect and function of public health research. Public health research is critical in the area of adolescent sexuality, reproduction, pregnancy and parenting to drive clinical and operational innovations in health care [18]. The study presented in this paper takes into consideration the public health perspective of sexual risk behaviour. Adolescent lifestyles and behaviour can be conceptualised using Brofenbrenner’s ecological model which is popular in public health research [43]. Within the Brofenbrenner’s model, behavioural risks can be analysed on fours levels: 1) microsystem (includes the immediate environment and socialization agents: family, peers, school and social media); 2) mesosystem (interactions between two microsystems); 3) exosystem (the indirect environment which includes the neighbourhood, health system, educational system) and 4) macrosystem (social values, cultural values, political and economic systems) [43].

It should be acknowledged that several scholars in South Africa have published studies on the relationship dynamics around adolescent pregnancy but most of these studies have been conducted in urban settings [32, 35, 36, 42]. According to Mkwananzi, young South African girls between the ages of 15 and 19, who are poor, from rural settings, and have low education levels are more likely to experience early childbearing [44]. The purpose of this paper is to elucidate adolescent mothers’ understanding of sexual risk behaviour, and thus the research question that is addressed is**: How do adolescent mothers understand sexual risk?** This paper will also seek to fill the gap in the literature by presenting the perspectives of sexual risk behaviour among adolescent mothers in rural, southern KwaZulu-Natal.

## Methods

### Study design

This paper emerged from a mixed-methods action research doctoral study (MMAR) that focused on the design of a community of practice model for a multidisciplinary and comprehensive approach towards caring for pregnant adolescents and adolescent mothers. The findings that are reported relate to the qualitative strand of the MMAR study and explored adolescent mothers’ understanding of sexual risk behaviour. The point of departure of the study was that adolescent childbearing is associated with unsafe sexual behaviour. By tradition, quantitative methods steered investigations into sexual risk behaviour among adolescents in the field of sexual and reproductive health. However, this qualitative strand of the study yielded thick and rich data and it was thus an effective portal for understanding the experiences of sexual risk behaviour by adolescent women. An explorative and descriptive qualitative study design was thus employed.

### Study setting

The study was conducted in a district hospital located in Ugu, KwaZulu-Natal, South Africa. The province of KwaZulu-Natal is the epicentre of the HIV epidemic in South Africa with a prevalence of 12.2% [30]. The high burden of HIV in KwaZulu Natal disproportionately affects women and adolescent girls because the economic, social, cultural and behavioural factors [45]. Ugu district is largely rural with an HIV antenatal prevalence of 41% [46]. Twenty-three percent of the deliveries at this district hospital involves adolescent girls between 13 and 19 years of age [47].

### Study population, sampling and sample size

The population of interest for this qualitative inquiry were adolescent mothers in the age category 13–19 years. For the purpose of this study, the definition of a first time adolescent mother will apply, namely a female between 13 and 19 years of age who has given birth for the first time to a live infant. The definition of adolescent mothers with repeat pregnancies for this study were individuals between 13 and 19 years of age who have given birth twice or more to live infants within 24 months. The participants were selected purposefully from this population and they were recruited during the quantitative strand of the larger doctoral study using a participant selection model. The participants were selected on the basis of meeting the criteria of the definitions of first time and adolescent repeat mother. Hence, the participants had experienced the phenomenon of adolescent pregnancy and motherhood. The comprehensive details of the quantitative strand of the doctoral study are described elsewhere [18]. Data were collected from 18 participants during four focus group discussions.

### Data collection

Focus group discussions were conducted to explore the selected participants’ understanding of sexual risk. The discussions were conducted during March and April in 2018. The learning resource centre at the hospital was used as a venue because the participants felt comfortable and safe there. These discussions were used to elicit the perceptions, ideas, opinions and thoughts of participants who had authentic experiences of the phenomenon under study [48]. Focus group discussions are also cost-effective [49]. The discussions were conducted in IsiZulu and audio recorded with the consent of the participants. A research team using a focus group discussion guide facilitated the discussions that ranged from 120 to 180 min each and four to five participants attended each of the four focus group discussions.

### Data analysis

Certain measures were put in place to ensure the authenticity and thus the trustworthiness of the data. The audio recorded data were transcribed verbatim and translated into English by a proficient research assistant. Thematic analysis was employed to code the data and categorise them into themes [49]. After the transcription of the recordings, the transcripts were carefully read and reread to become familiar with the data. The researchers further ensured the trustworthiness of the data by sharing the transcripts and findings with the research supervisors to ensure that the data had been analysed correctly and appropriately [49]. The research team was familiar with the research site and the participants prior to data collection. The advantage of being familiar with the research site and participants enhanced rapport and communication during the study. According to Hockey [50], researching in familiar settings increases the likelihood of participants revealing more intimate details of their lives. The transcribed data were referred to the participants for review. A thick, detailed description of the methodology that was employed will ensure the reproducibility of the study [51].

### Ethical considerations

The University of KwaZulu-Natal Bioethics Research Committee (ref no: BFC553/16), the KwaZulu-Natal Department of Health (ref no: KZ_2016RP26_545), and the Chief Executive Officer of the district hospital approved this study. Participation was voluntary and the participants could withdraw from the study at any point. Written consent was granted by all participants. Parents’ or legal guardians’ permission was obtained for each participant under the age of 18 who participated in the study.

Pseudonyms were used to identify the illustrative quotes by the participants and to ensure that their confidentiality is maintained.

## Results

The socio-demographic characteristics of the participants are presented in Table 1. The participants were 18 adolescent mothers in the 16–19-year age group. Seventeen of the participants experienced unplanned pregnancies. Of the eighteen participants, 13 had dropped out of school due to their pregnancy. Nine of the eighteen participants were adolescent mothers who had experienced repeat pregnancies Seven overarching themes emerged from the focus group discussions: factors that contribute to sexual risk behaviour, culture versus sexual risk taking, gender, power and sexuality, perceptions of love and romantic relationships, reproductive health services, preventing a repeat adolescent pregnancy, and plans regarding the next pregnancy and birth control.

**Table 1:** Socio-demographic characteristics of the participants (*N* = 18)

Characteristics	Number of participants (Percentage)
Age	
16–17	5 (28%)
18–19	13 (72%)
Number of pregnancies	
1	9 (50%)
2	9 (50%)
Planned/Unplanned Pregnancies	
Planned pregnancies	1 (5.6)%
Unplanned pregnancies	17 (94.4%)
Living arrangements	
Living with biological mother	3 (16.6%)
Living with biological parents	1 (5.6%)
Living with biological mother and siblings	5 (27.8%)
Living with biological parents and siblings	1 (5.6%)
Living with maternal grandparents	2 (11.1%)
Living with biological sister	3 (16.6%)
Living with an aunt	2 (11.1%)
Living with aunt and siblings	1 (5.6%)
Current relationship status	
In a relationship/not living together	12 (66.6%)
Engaged	1 (5.6%)
Single	5 (27.8%)
Education Level	
Grade 7	1 (5.6%)
Grade 10	6 (33.3%)
Grade 11	7 (38.9%)
Grade 12	4 (22.2%)
Schooling status	
Completed schooling	4 (22.2%)
Dropped out of school	13 (72.2%)
Resumed schooling	1 (5.6%)
Employment status	
Employed	0
Unemployed	18 (100%)

### Theme 1: factors that contribute to sexual risk behaviour

Under the overarching theme of factors that contribute to sexual risk behaviour, the participants identified peer pressure, drugs and alcohol, sexual experimentation, myths about contraception, unprotected sex, the media, parental influence, poverty and transactional sex, vulnerability, and partner rejection as factors that provoke adolescent pregnancies.

#### Peer pressure

According to the illustrative quotes of the participants, engaging in sexual intercourse has become a norm if young girls want to be accepted by their peers. Two of the participants, Olwethu and Maya, recollected that their peers were critical of virgins. Maya and Mary believed that engaging in sex was similar to being part of a movement or trend, and it made them ‘acceptable’:

“All young people are engaging in sex and nobody wants to be an outcast” (Smangele, 18).

“Peer pressure is causing adolescents to have sex. Adolescents think that they have to keep up with trends and behave like everyone else. If you are a virgin, others will tease you” (Olwethu, 18).

“Often, sex is treated like a fashion trend among our peers. I remember being teased when my friends found out that I was still a virgin and I had a boyfriend. My boyfriend was also being teased by his friends” (Maya, 18).

“Adolescents are ignorant and want to engage in sex because of peer pressure” (Sbahle, 18).

“Puberty causes adolescents to become restless and they are easily manipulated by peer pressure” (Palesa, 18).

“Sex has become a fashion for adolescents. Peer pressure also influences sexual behaviour” (Mary, 18).

#### Drugs and alcohol

Adolescent girls under the influence of alcohol and drugs are vulnerable and engage in risky sexual behaviour. Palesa understood that unprotected sex is associated with the risk of pregnancy and HIV infection:

“Drugs and alcohol promote risky sexual behaviour. The girls are drunk at parties and they are unaware of their actions” (Palesa, 18).

“Adolescents are using drugs and alcohol at parties and this puts them at risk for pregnancy and HIV infection. In the drunken and drugged state, they have unprotected sex” (Sphe, 16).

#### Sexual experimentation

The participants confirmed that adolescents are at a stage in their lives when they want to experiment with their bodies. Smangele compared sexual intercourse to an experiment. Snothando also suggested that adolescents were keen to experiment with sex, drugs and alcohol and they remained oblivious to the negative consequences.

“Adolescents want to experiment and therefore indulge in risky behaviours. In my community, adolescents are fond of keeping up with new trends and they are willing to experiment with their bodies. Sexual intercourse is an experiment for young people” (Smangele, 18).

“It is all about ignorance. Adolescents girls do not think that bad things will happen to them and they will experiment with boys. I think it is the hormones in the adolescents that make them experiment with sex, drugs and alcohol” (Snothando, 19).

“Once you have a boyfriend, you will engage in sexual activities. Boys convince girls to have sex. It is how boys want girls to show their love” (Olwethu, 18).

#### Myths about contraception, partners’ perceptions, and unprotected sex

The participants narrated their concerns about contraception and how this resulted in their engagement in unprotected sex. Many were concerned that contraception caused sterility. Some mentioned that their partners did not like using condoms.

“My partner was not fond of using condoms and I was not on contraception. I believe that contraception makes you fat and sterile. So we were having unprotected sex” (Smangele, 18).

“My partner and I didn’t use protection. I was told by many people that contraception causes problems with fertility and as an adolescent you must not use contraception. As a young woman, one must have a baby first to test fertility and show the ability to conceive” (Thobeka, 17).

“We didn’t use protection. I believed that contraception causes infertility in adolescents. It is important for a woman to prove her fertility because no one will marry her [if she doesn’t]” (Joyful, 18).

“I did not believe in contraception because it causes complications. I believe the biggest complication of contraception is sterility. Other young women also do not trust contraceptive methods because it damages the body. The female condom is also uncomfortable and men do not like using condoms because it reduces pleasure” (Nana, 19).

“Adolescents do not trust any of the contraceptive methods because they still fall pregnant even if they use them” (Amahle, 17).

“I was on the injectable contraceptive. I was scared of infertility and weight gain so I skipped the injection. He didn’t use a condom because he believed it was my responsibility to prevent a pregnancy” (Jenny, 19).

“At first, I used the injection method but later I experienced problems, and I stopped the contraception. My boyfriend never uses condoms. It feels embarrassing to tell a man to use a condom” (Olwethu, 18).

#### The media

The participants believed that the media had a significant role in promoting high risk sexual behaviour. Yvonne narrated that sex was explicitly portrayed on television and that it was thus condoned.

“The media shows us that it is okay to have sex and there is no shame in premarital sex” (Snothando, 19).

“Sex is even in the media. We watch programs that encourage physical love. The local programs on television do not condemn sexual relations outside marriage so I do not know why people in the community are judgmental. We have to keep with current trends” (Yvonne, 16).

#### Parental influence, inappropriate behaviour of parents and children’s reaction

In this subtheme, parental influence and inappropriate behaviour of parents were expressed by participants. Sbahle stated that parents exposed their children to sex while Tholothando mentioned that lack of parental supervision also influenced the sexual behaviour of children. In this regard, parental influence and inappropriate behaviour of parents were expressed by participants. Amahle narrated an incident in which her mother had accused her of engaging in sexual intercourse. Angry about this false accusation, Amahle decided to engage in sexual intercourse to retaliate against her mother. The quotes also illustrate how children react to their parents behaviour.

“Parents are having sex in front of their children. Parents also have sex with their own children” (Sbahle, 18).

“It depends on the rules in the house and how parents treat their children. If you have freedom to have a boyfriend, then you will have sex with him before marriage” (Tholothando, 19).

“In my situation, my mother accused me of sleeping around and I decided to prove her right. I was feeling so aggressive. So I decided to sleep around. Parents anger their children. When relationships are not good at home, this will cause children to look for comfort elsewhere” (Amahle, 17).

#### Poverty and transactional sex

The participants indicated that poverty resulted in transactional sex. Older men were identified as perpetrators who took advantage of younger women in need of financial help. These older men were referred to as ‘blessers’. The participants stated that adolescent girls returned the financial favours by engaging in sex with these older men. Amahle mentioned that being involved with a ‘blesser’ had become the norm as adolescent girls were encouraged to have relationships with older men for financial gain.

“If the girl’s family is poor, then she will look for financial help from a man. There are ‘blessers’ who will help you but you have to sleep with them” (Tholothando, 19).

“Adolescent girls get involved with older men for financial reasons. Poverty drives transactional sex” (Nana, 19).

“Older men seduce and manipulate young women. If you are from a poverty-stricken home, an older man is a blesser. He provides you with money and gifts” (Joyful, 18)

“Girls are encouraged to find an older man to help them financially. In return, the girl has sex to please him” (Amahle, 17)

“If you receive gifts then you will want to thank the man by pleasing him. The rich man will also continue to shower you with gifts if you are having sexual relations” (Snothando, 19).

.

“Poverty and poor family background cause adolescents to engage in transactional sex with older men” (Palesa, 18).

#### Vulnerability and the fear of partner rejection

Some participants referred to the vulnerability of adolescent women and stated that fear of rejection by their partners compelled them to engage in sex to maintain their relationships. Joyful clearly stated that adolescent women gave in to sexual demands to avoid rejection.

“Young women have premarital and unprotected sex to please their partners as they fear rejection” (Harriet, 17).

“Young girls are vulnerable and they search for love and attention. Boys manipulate girls and girls start engaging in sex to prove they are attractive. Girls also give in to sexual demands because they fear rejection” (Joyful, 18).

### Theme 2: culture versus sexual behaviour

With reference to the theme of culture versus sexual behaviour, the participants understood that culture discourages premarital sex. The participants stated that virginity testing was a traditional practice and that their culture encouraged young people to be proud of their virginity. Furthermore, they stated that their culture encouraged the preservation of virginity until marriage.

#### Culture condemns premarital sex

Culture may be associated with taboos about sexuality and it may have an impact on the practice of sexual and reproductive health. The participants in this study expressed the prescriptions that are set out by their culture.

“Culture influences the sexual behaviour of teenage girls, for example the reed dance. Virginity testing is an important traditional practice. Young girls may want to conform to what culture teaches you about sex. Culture does not promote premarital sex” (Harriet, 17).

“My culture advises young girls not to have sex with men until they are married” (Mary, 19).

“Culture advises us to be proud of our virginity and wait until marriage to have a baby” (Jenny, 19).

#### Individual preference disregards the cultural rule of abstinence

Some participants acknowledged that a changing lifestyle had encouraged opposition to cultural rules regarding sexual behaviour, and modern young people’s individual preferences dominate over cultural practices. One adolescent mother admitted that tampering occurs when virginity testing is done due to bribery. Another participant mentioned that adolescent girls engaged in premarital sex because lobola (Lobola is a dowry payment by the bridegroom to the bride’s family in most African countries) was a lengthy process.

*“*Well, in our culture, to remain a virgin until marriage is considered a good thing, but peer pressure and a change in lifestyle are influencing adolescent girls to have sex. The reed dance custom doesn’t work anymore because adolescent girls bribe the woman who is doing the testing for a good result. This means the woman testing for virginity will lie by saying that a girl is a virgin because she has been bribed” (Snothando, 19).

“We understand that our culture says we must wait for marriage and then have a baby, but people today have different preferences” (Sbahle, 18).

“Many adolescent girls have sex early because they do not want to wait too long for the lobola process. They know the cultural rules but they are influenced by the opinion of others and their own preferences” (Tholothando, 19).

### Theme 3: gender, power and sexuality

Most of the participants acknowledged that a combination of gender, power and sexuality influenced sexual risk behaviour among adolescent women. Gender inequality was referred to as a common feature in the community, as the participants argued that adolescent women were not respected in society and they were thus disempowered. Society was perceived as an institution that had double standards regarding the sexual behaviour of men and women.

#### Gender inequality

Gender norms including inequality also has a huge impact on the sexual and reproductive health of adolescent women. Gender inequality compounds the discrimination faced by adolescent girls. The quotes illustrate the vulnerabilities of adolescent girls to gender inequality.

*“*There is no equality between boys and girls in my community. Boys are disrespectful. They treat girls as sexual objects. The boy makes all the rules for the relationship and demands a sexual relationship” (Harriet, 17).

“Boys treat girls in a bad manner. They do not respect girls and women. There is no equality between boys and girls. Boys think they are superior. The community favours boys. Only girls are cursed if they fall pregnant before marriage but it is okay for boys to have children before marriage. This allows boys to exploit girls sexually” (Olwethu, 18).

“Boys are disrespectful and abusive towards girls. Our community is to blame for the inequality between boys and girls. Girls are ill-treated for getting pregnant while boys escape the taunts and discrimination. Girls are played with, abused and thrown away” (Palesa, 18).

#### Objects of pleasure

According to the participants, young women are mere sexual objects or commodities for men that can be purchased and discarded. Amahle [17] stated that “Men view women as the weaker gender. They think that women are sexual objects or toys that can be bought”. Sbahle [18] expressed that women experience a culture of disrespect. She stated that *“*Boys do not treat girls well. They feel girls are like toys. You can play with toys and throw them away”. Mary [19] reiterated the culture of disrespect by stating that “These men in my community treat women like cheap objects who can be bought and sold easily”.

#### Exploitation

The participants spoke intensely of the sexual exploitation of young women in their communities. One participant perceived society as undermining equality between men and women, and she argued that this was a driver of exploitation.

*“*Most of the girls are alcoholics in my community and the boys take advantage of them. The boys know that these girls are easy targets for sex. They do not respect girls in general” (Joyful, 18).

“Young girls are always exploited by older men because of poverty. In our communities, rules are different for men. Society places more emphasis on women maintaining a proper image in society. This leads to discrimination against women. We are seen as a weaker sex that should be dominated by men” (Maya, 18).

### Theme 4: adolescents’ perceptions of love and romantic relationships

This theme illuminated the participants’ perception of love and romantic relationships. According to the participants**,** love and romantic relationships should encompass respect and emotional and financial support and they perceived loving partners as nurturers and providers. One of the participant also felt that love is also conveyed or extended when a man who takes the responsibility for paying lobola (a bridal dowry) for his partner.

#### Respect, emotional and financial support

The participants expressed that respect, emotional and financial support were important aspects to love and romantic relationships.

“Respect is an important aspect of love and romantic relationships. I think that love for me means that my partner will respect me and also take responsibility for me emotionally and financially. He will pay lobola for me” (Amahle, 17).

“Love and romantic relationships are about mutual respect and receiving attention and kindness. A loving partner will take care of you emotionally and financially. He will always provide for your needs” (Sphe, 16).

“Love is about respect and support. Love means that a man should also provide for the woman. He should support her financially” (Sbahle, 18).

#### Physical expressions of love

The participants outlined the importance of physical expressions of love in their perception of love and romantic relationships. Puleng [18] stated that “Love is about being comfortable with your partner. It is about the physical connection and chemistry in the relationship” Physical expressions of love encompassed not only physical gestures like hugging, but also the provision of lavish gifts. Joyful [18] articulated “Love and romance is about physical gestures like holding hands, kissing and spending lavishly on gifts. Love needs to be shown actively”.

### Theme 5: reproductive health services

Within the theme of reproductive health services, attention is drawn to the following subthemes: the judgmental attitude of healthcare workers and a shortage of injectable contraceptives. The participants explained how reproductive health services influenced their sexual health seeking behaviour. Some participants reflected on the judgmental attitudes of healthcare workers when they tried to access family planning services.

#### The judgmental attitude of healthcare workers

The participants also perceived discrimination in the healthcare settings. The participants expressed that healthcare workers were not supporting adolescent girls access to sexual and reproductive health services.

“The nurses are judgmental. They treat you badly if you ask about family planning services. They had refused to give me an injectable contraceptive before I fell pregnant because I was apparently too young” (Thobeka, 17).

“Nurses are judgmental because they believe we are using contraception to avoid falling pregnant. They are not happy that we are sexually active” (Tholothando, 19).

“The clinics are not adolescent friendly. Nurses make fun of adolescent mothers who want to access contraception. The queues are long and nurses are slow” (Snothando, 19).

#### A shortage of injectable contraception at clinics

In addition to their general concerns about reproductive health services, the participants referred to a shortage of injectable contraceptives at the clinics. Nana [19] stated that “At times, there is a shortage of injectable contraceptives at the clinic” Palesa [18] stated that “There is sometimes no injectable contraceptive available at the local clinic. I usually return home without receiving the injectable contraceptive. The shortage of injectable contraceptives compounds the unmet need for contraception and poses a risk for a repeat pregnancy, especially to adolescents that use this method of contraception.

### Theme 6: advice on preventing a repeat adolescent pregnancy

Under the theme of advice for preventing a repeat pregnancy, three subthemes were identified: contraception and condoms, abstinence, and sceptics. Some participants emphasised the importance of using contraception and condoms.

#### Contraception and condoms

The participants advised that contraception and condoms should be used to prevent a repeat adolescent pregnancy. The advice on the use of contraception and condoms in the prevention of repeat adolescent pregnancies is echoed the following quotes.

“I would encourage other adolescent mothers to use contraception and condoms to prevent a repeat adolescent pregnancy. This means the use of both male and female condoms” (Sphe, 16).

“I think protection during sexual intercourse is very important to prevent a first and repeat pregnancy. The protection should include contraception and condoms” (Puleng, 18).

#### Abstinence

The participants who had experienced repeat pregnancies felt tangible regret and remorse. They promoted abstinence as a strategy to prevent a repeat pregnancy. The support for abstinence as a measure to prevent adolescent repeat pregnancy is well articulated in the following quotes:

*“*I experienced a repeat pregnancy. This made life very difficult so I recommend abstinence to other first time mothers. After the first pregnancy, she should abstain [from having sex] and concentrate on her studies” (Mary, 19).

My advice would be to delay sexual intercourse. At the age of 18 years, I have two children and our future is uncertain. I would encourage first time mothers to abstain and concentrate on completing their high school education (Maya, 18).

#### Sceptics

Some adolescents expressed uncertainty about using contraception and condoms to prevent a repeat pregnancy. Amahle made reference to men being able to alter the effects of contraception by using traditional medicine when they impregnate a woman, while Harriet did not trust condoms and contraception.

“I do not think a girl can prevent a repeat pregnancy because men will use traditional medication [herbs] to make a girl fall pregnant even if she is on contraception” (Amahle, 17).

“I do not trust contraception and condoms. Abstinence is not an option for adolescent mothers. Maybe you can use the morning after pill” (Harriet, 17).

### Theme 7: plans for another pregnancy and birth control

The participants shared their plans regarding a next pregnancy. Many did not want another pregnancy and would use the injectable contraceptive to prevent falling pregnant. The following quotes echoes the sentiments shared by adolescent mothers on future plans regarding childbearing and the use of birth control:

#### Preference for an injectable contraception

Most participants were in favour of the long acting injectable contraception which is a form of contraception that is injected into the muscle.

“I am not going to have another baby any time soon. I want to use the 3-month injectable contraceptive” (Thobeka, 17).

“I am not having any more children. I will use the injectable contraceptive and encourage my partner to use a condom” (Puleng, 18).

“I am not planning to have another baby till I am financially stable. I am currently using the injectable contraceptive” (Yvonne, 16).

“I am using the 3-month injectable contraceptive and I will not have another baby soon. I will complete my high school education” (Thobile, 19).

#### Reluctance to use birth control

Some participants expressed a lack of faith in contraception and they had not decided on a birth control method to prevent another pregnancy. The participants also articulated reasons as to why they were reluctant to use contraception. The following quotes illustrates the reluctance of participants to use birth control.

*“*I do not plan to have another baby. I won’t use long acting contraception or the pill because it makes you fat, damages your skin and makes you look ugly. I will use the morning after pill” (Harriet, 17).

“Another pregnancy is out of the question! I have no faith in contraception. I have not decided about my future plans regarding birth control” (Olwethu, 18).

“I do not want to have more children in the future. I have too much responsibility with my two children. I have lost faith in contraception. I would like to do a ligation” (Sbahle, 18).

## Discussion

The purpose of this study was to explore and better understand adolescent mothers’ perceptions of sexual risk behaviour. Following data collection and analysis, deep insight was elicited about the sexual health decisions that adolescent mothers took after their first pregnancy. The seven main themes that will steer the discussion are: factors that contribute to sexual risk behaviour, traditions (culture) versus sexual risk taking, gender power and sexuality, perceptions of love and romantic relationships, reproductive health services, prevention to avoid a repeat adolescent pregnancy, and plans regarding a next pregnancy and birth control.

The current study found that peer pressure, drugs and alcohol, experimentation, myths about contraception influenced or shaped adolescents sexual risk behaviour. Furthermore, the media, poor parental supervision and power gender dynamics, poverty leading to transactional sex, the vulnerability of young girls, and the fear of partner rejection, also contributed to the risky sexual behaviour of adolescents. The sexual risk behaviour of adolescents is connected to environmental influences. Social agents like the family, school, peers and social media in the microsystem (immediate environment) played a role in contributing to risky sexual behaviour.

Prior studies have noted the impact of peer pressure that prompts adolescents to engage in risky sexual behaviour [1, 19, 20, 46, 52]. Some of the adolescent mothers in this study stated that engaging in sex had become the norm and was a ‘fashion trend’. A study by Phaswana-Mafuya et al. [22] also found that adolescents and adults viewed adolescent pregnancy as normative and fashionable and thus the ‘in thing’ to do. Clearly, adolescents in this district are highly likely to engage in risky sexual behaviour based on the belief that their peers are involved in similar behaviour, which suggests that adolescents model the viewpoint and behaviour of their peers rather than the traditional values of society.

Myths about contraception also influence sexual health practices [17, 53], as the beliefs that the participants held about contraception shaped their health practices and their sexual risk taking behaviours. This finding concurs with a study by Ngum Chi Watts et al. [17] that was conducted among Australian adolescent mothers who also believed the myth that contraception causes sterility. Adolescent mothers in both studies reported a distrust in contraception because they had witnessed adolescent women falling pregnant regardless of contraception. The overwhelming fear of weight gain while using contraception was echoed by our participants and also by Brazilian women in a study conducted by Goncalves et al. [53]

The participants in our study also noted the negative attitudes of their partners towards condoms, which compromises safe sex. The disfavour of South African men towards condoms are noted by Mash et al. [54] Likewise, Kanda and Mash [55] found that Batswana men and women in their study believed that condoms reduces pleasure, and interferes with a sustained erection. Gendered norms also place the responsibility of contraception in the women’s domain. An adolescent father in the South African study Chili and Maharaj [56] articulated that the contraceptive methods were solely his choice and sex without a condom is a display of masculine power. Male power domination also results in unsafe sexual practices, unplanned and unwanted pregnancies. Furthermore, inconsistent condom use and partners’ repudiation of safe sexual practices hamper public health efforts to promote HIV protective behaviours [54].

Alcohol and drug use (or abuse) undeniably impairs an individual’s judgement and influences risky sexual behaviour [57]. The role of drugs and alcohol in promoting risky sexual behaviour has also been noted by Jackson et al. [23], who analysed the data from two cohorts of Scottish adolescents to determine the impact of substance use (illicit drugs, smoking and alcohol consumption) on sexual behaviour. It was noted that drugs and alcohol increased risky sexual behaviour in the participating adolescents [23]. Puberty is a landmark period for the development of sexuality in adolescents and they are likely to engage in sexual experimentation during this period [58]. This was also a trend among the participants in the current study. Sexual curiosity which is associated with psycho-emotional factors is common among adolescents as they are learning to explore and understand sexuality. Adolescents are adjusting to their social needs, changes of their bodies and sexuality [43]. Adolescent mothers in Ghana confirmed that adolescence is a rebellious phase and they admitted that they wanted to experiment by engaging in sex [59]. In most instances, experimentation includes substance use (drugs and alcohol) and sex [57].

In the current study, the participants also referred to the sexually explicit content that appears in the media. They agreed that this phenomenon influences their sexual attitudes, beliefs and behaviour. According to Nagaddya et al. [60], social media also facilitates risky sexual behaviour through unsolicited (and often solicited) photos, videos and texts. Adolescents who were interviewed in a Ugandan study on the impact of social media content and sexual behaviour reported that sexual content posted on social media caused a definite change in their sexual behaviour, including a lively interest in engaging in premarital sex and masturbation [60]. Similarly, the participants in our study felt that society should not condemn premarital sex as it is shown explicitly and thus condoned in television programs. Television is avidly viewed by adolescents and thus provides inviting observational effects of sexual activities. Oladeji and Ayangunna [61] and L’Engle et al. [62] argue that adolescents who are consistently exposed to sexual content on the media, and who perceive this sexual content positively, are more likely to engage in risky sexual activities than those who are not exposed to such material.

The participants in this study revealed that some parents’ behaviour encourages adolescents’ risky sexual activities. Some mentioned that children were exposed to their parents engaging in sex while some mentioned that a lack of parental supervision gave adolescents the liberty to engage in risky sexual behaviour. Participants also revealed that some fathers engaged in sexual activities with their daughters. According to Landry et al. [63], empirical evidence has shown that parental supervision and monitoring are usually protective factors that curb inappropriate sexual risk behaviour. Moreover, the current study revealed that poor parental relationships could also result in risky sexual behaviour, as some participants admitted that their difficult relationships with their parents resulted in their rebellious behaviour. Earlier studies also found that dysfunctional mother-daughter relationships resulted in risky sexual behaviour that led to adolescent pregnancies [1, 21, 64].

The participants acknowledged that poverty and the lure of transactional sex were also reasons for risky sexual behaviour, as many young women from poverty-stricken homes would be encouraged by older men to engage in a sexual relationship as they would provide for them financially. The promise of expensive gifts was also mentioned as a powerful enticement. In recent years, there has been an increasing interest in the ‘blesser and blessee’ phenomenon as a driver of the HIV epidemic [30]. Older, rich men are known as ‘blessers’ who tempt young women (‘blessees’) by providing them with money and gifts for sexual favours [28]. According to the participants in the current study, sex is transacted for money and gifts by older men. A study in Wentworth, South Africa, revealed that both adolescent girls and boys were lured into transactional sex [65]. A qualitative study by Raganathan et al. [66] in rural Bushbuckridge in Mpumulanga, South Africa, also found that young women perceived transactional sex as a way of securing financial success. The women from the study in Mpumulanga also viewed money and gifts as a determinant of a woman’s value and the commitment of the man to her [66]. Poverty in a larger context within the macrosystem has a cascading effect on an individual’s behaviour.

However, the perception of transactional sex as ‘a blessing’ undermines public health efforts to prevent HIV and STIs in both rural and urban communities in South Africa [31, 66–68]. In a Ghanaian study by Kyilleh et al. [69],adolescent women also reported that poverty prompted young women to engage in transactional sex. It is noteworthy that in the latter study the male participants reported that adolescent school-going girls had multiple relationships in order to sustain themselves financially [68].

It was evident from the findings of our study that the participants’ vulnerability and the fear of rejection encouraged their risky sexual behaviour. Similarly, Hafen et al. [70] found that adolescents are sensitive and fearful of partner rejection. For an adolescent woman, rejection by a partner embodies anxiety and emotional turmoil [70]. In this regards, the fear of partner rejection makes adolescent women feel vulnerable and, in order to save a relationship, they are most likely to engage in risky sexual behaviour [69].

The literature on young women’s perception of how culture influences their sexual behaviour is limited, and a question pertaining to this theme was posed to address this gap. The ecological theory explains that culture within the microsystem and macrosystem influences the development of an individual [43]. Commenting on culture, Nelson, Morrison and Breedy [71] argue that culture is beyond race, ethnicity, gender and age, and this is an important stance when discussing culture and sexual risk factors. For example, all the participants in our study belonged to the Zulu ethnic group and they all admitted that their culture placed a high value on the preservation of their virginity. Guiso et al. [72] define culture as “customary beliefs and values that ethnic, religious and social groups transmit fairly unchanged from generation to generation” (p. 23). Virginity testing is a traditional practice in the Zulu culture [73]. In this context, the participants acknowledged the role of culture in issues of sexuality but also underscored the changing values of modern adolescents. Chisale [73] points out that cultural practices are under threat as a result of criticism from feminist movements and human rights organisations [1], and thus virginity testing has received a backlash from human rights and gender activists as a violation of a woman’s sexuality and her rights [54]. However, in response, Chisale [73] argues that virginity testing is tantamount to “feminine power over patriarchy” (p. 225), as it is a form of protection.

Some participants in our study also reported that the payment of lobola for a bride, which is a traditional Zulu practice, is a lengthy process and some young women are unwilling to wait for its conclusion. According to Posel and Rudwick [74], Zulu adults find lobola a constraint to marriage due to the high costs. Lobola is a form of compensation that the bridegroom pays to the bride’s family for their loss of a daughter, and it is also a symbol that unites the two families [75]. The payment was traditionally done in the form of cattle, but large sums of money have also been accepted more recently. The payment of lobola is not without cultural value, but it has been criticised by the Western world and by women’s groups and lobbyists in South Africa as a form of purchasing and trading in women [72]. Cultural practices in the microsystem and macrosystem are often plagued by freedom in the Western societies [43]. Current debates about cultural practices are important, especially as they address changing sexual practices. Moreover, the debate about individualism versus culture must not be ignored, particularly as individual preferences now marginalise cultural rules about abstaining from premarital sex. This was clearly illustrated by the comments of the participants in the current study.

Since the dawn of democracy, South Africa has remained a patriarchal society where adolescent pregnancy is viewed as a form of delinquency and even prostitution [76]. Moreover, Hodes [76] argues that some South African males oppose gender equality as they view it as a license for sexual promiscuity among adolescent women. In the current study, the participants were disconcerted by the taunts of society when they became adolescent mothers. They also commented avidly on a persisting culture of disrespect and gender inequality. Research in South Africa has shown that the social contexts also influence and shape the experiences of adolescent sexuality and women’s subordination [35]. Furthermore, adolescent pregnancy in South Africa is met with social disapproval. Botha [35] suggests that communities associate adolescent pregnancy with moral degeneration. Political and social discourses at a macrosystem level can either affect the individual positively or negatively [43]. Hodes [76] argues that the attitudes dominating adolescent pregnancy violates woman rights.

While the literature suggests that some adolescent women are intentionally becoming pregnant for material gain by exploiting the child support grant [77, 78], the participants in our study focused on the sexual exploitation of poverty stricken adolescents by older men. Gender inequality and the power struggle in a relationship compromise the sexual and reproductive health of adolescent women [79]. Dominant and controlling male partners are likely to engage in risky sexual behaviour and are highly likely to infect their partners with HIV/STIs [80]. The power imbalance in the relationship of an adolescent woman with an older man or an abusive young man also renders her vulnerable to predatory sexual advances and/or risky sexual behaviour [79].

Conversely, the participants in our study associated love and romance with respect, emotional support, financial support and physical expression. Sumter et al. [80] also state that adolescents perceive love and romance as encompassing passion, connection and physical intimacy. However, the literature suggests that love in the context of romantic relationships often renders adolescents vulnerable and encourages them to engage in sexual activities [69, 81]. Furthermore, adolescents may underestimate the sexual risks they take and are highly likely to have unprotected sex. A study that was conducted in India by Janardhana and Manjula [81] found that adolescent women who engaged in physical intimacy and premarital sex with a romantic partner thought that it would lead to a marriage proposal. A similar tendency was noted by Kyilleh et al. [69], as Ghanaian adolescent women engaged in premarital sex with the expectation that their partners would marry them. This study affirmed that an in-depth understanding of love and romantic relationships is pivotal in the context of adolescent sexual and reproductive health, with particular reference to pregnancies, HIV, and STIs.

The participants in this study reported that they were concerned about the judgmental attitude of nurses and limited stocks of injectable contraceptives at clinics. Furthermore, participants in this study did not consider the clinics to be adolescent friendly. Healthcare services is a part of the exosystem that has a profound effect on the development and behaviour of individuals. A systematic review of the attitudes of healthcare providers towards adolescent sexual and reproductive health services in developing countries found that unprofessional attitudes of healthcare professional and a lack of youth friendly reproductive health services hindered access to sexual and reproductive health services [82]. According to local research, Mkhwananzi also noted that South African adolescent mothers are subjected to taunting from nurses [83]. Furthermore, studies conducted in Ethiopia [84] and Thailand [85] also found that the negative attitude of healthcare practitioners reduced the young girls’ desire to access sexual and reproductive health services. This suggests that healthcare services may be a barrier to rather than an enabler of sexual and reproductive health support. In our study, the perceptions of the participants illustrate that sexual and reproductive health is provided in a demeaning manner rather than in an empowering manner. In this regard, the exosystem can be demeaning or empowering to the individual [43].

The participants in this study proposed contraception, condoms and abstinence for the prevention of an adolescent repeat pregnancy. However, some participants were skeptical about the advice they had received regarding the prevention of a repeat pregnancy, which was probably due to the myths surrounding contraception and the fear of weight gain among adolescent women. There is limited literature on the postpartum sexual and reproductive health plans of adolescent mothers. This is a matter of grave concern because the sexual and reproductive health needs of adolescent mothers are often shelved due to a lack of understanding and knowledge. Most of the participants in our study were adamant that they did not want to have another baby and many stated that they preferred the injectable contraception. Some were keen to encourage their partners to use a condom as well. However, some participants had no plans regarding the use of contraception due to their distrust of the efficacy and side effects of contraception. Studies in Australia [13] and Brazil [53] also provide evidence that young women have many misconceptions and fears regarding the use of contraception.

This study had several limitations. The sample size was small and it was conducted in only one health establishment in the Ugu district. The transferability of the results is therefore limited to a setting that is similar to the study site. The study was also confined to adolescent women while the views of adolescent young men were not elicited. It is acknowledged that the sexual risk behaviours of adolescent men are equally important when considering recommendations for improving sexual and reproductive health behaviours and services, and this may become the focus of a future study.

## Conclusion

The findings of this study contribute to an in-depth understanding of adolescent mothers’ perspectives of sexual risk behaviour. In view of our analysis of the data, it may be argued that adolescent women’s beliefs about their family and friends, love and romantic relationships, contraception, and cultural practices and behaviours will affect their sexual and reproductive health practices. Similar to the findings of Yakubu and Salisa [86], the findings of this study also confirm that poor sociocultural, economic, individual, and health service related factors prompt inappropriate sexual behaviour among adolescents. In considering the sexual and reproductive health of adolescents, their right to make independent decisions and the impact of cultural impositions on these decisions should not be sidelined. One noteworthy finding is that adolescent women are vulnerable to transactional sex. Poverty drives them into inappropriate and harmful sexual relations with older men as this is often their only means of survival. Moreover, transactional sex is associated with HIV, STIs, adolescent pregnancy and sexual violence [66, 67] and should be curbed as a matter of urgency. The economic empowerment of adolescent girls including financial literacy is essential to combat poverty and transactional sex [87]. Gender and power inequality in adolescent romantic relationships also increases young women’s susceptibility to HIV infection [77].While transactional sex is a natural concern, adolescent mothers in this study had stated that the provision of lavish gifts is an important part of a loving relationship. In this regard, the nature of adolescent romantic relationships needs to be further explored.

The findings of the study reinforce the importance of postpartum sexual risk reduction interventions for adolescent mothers, with particular reference to HIV/AIDS and adolescent pregnancy. The authors have paved the argument that sexual risk behaviour must be understood in terms of public health issues. Sexual and reproductive health (SRH) education should be underpinned by resilience, consequential thinking, navigating emotions, the right to negotiate, and skills in decision making. South Africa is a signatory to the Sustainable Development Goals (SDGs) of the United Nations and SDG 3 in particular addresses health issues and focuses on ensuring universal access to SRH. This can only be achieved through transformative collaboration between the health, education and social sectors, and this should be encouraged in the interest of the many young people in this country who are victims of ignorance, poor decision making, and the need for survival.

## Data Availability

The data used to elicit the findings of this study are available from the corresponding author upon reasonable request.

## Abbreviations

HIV: Human immunodeficiency virus
AIDS: Acquired immunodeficiency syndrome
SDG: Sustainable development goal
SRH: Sexual and reproductive health
STI: Sexually transmitted disease
